# Effect of thymol on oxidative stress and reelin signaling pathway in Alzheimer’s disease model

**DOI:** 10.55730/1300-0152.2683

**Published:** 2024-02-01

**Authors:** Barış BİTMEZ, Burcu ÇEVRELİ, Emel KAŞIKÇI

**Affiliations:** 1 Department of Molecular Biology, Faculty of Engineering and Natural Sciences, Uskudar University, İstanbul, Turkiye; 2 Neuropsychopharmacology Research and Application Center, Uskudar Univesity, İstanbul, Turkiye

**Keywords:** Alzheimer’s disease, thymol, d-galactose, aluminum, reelin, LRP8

## Abstract

**Background/aim:**

The purpose of this study was to investigate how thymol affects cognitive functions and the levels of MDA, GSH, Aβ_1–42_, ApoE, reelin, and LRP8 in an AD model induced in male Wistar albino rats with the application of D-galactose (D-gal) and aluminum chloride (AlCl_3_).

**Materials and methods:**

In this work, 3-month-old male Wistar albino rats were used. Group 1 served as the Control, Group 2 received 0.5 mL/day saline + 0.5 mL/day sunflower oil, Group 3 was administered 200 mg/kg/day AlCl_3_ + 60 mg/kg/day D-gal, Group 4 received 30 mg/kg/day thymol, and Group 5 was administered 200 mg/kg/day AlCl_3_ + 60 mg/kg/day D-gal + 30 mg/kg/day thymol. At the end of the 10-week experimental period, behavioral and memory tests were performed. GSH and MDA levels were measured in the obtained serum and brain tissue samples, while Aβ_1–42_, ApoE, reelin, and LRP8 levels were measured in brain tissue samples. Statistical analyses were performed using ANOVA test in Graphpad Prism V8.3 program. A p-value <0.05 was considered significant in intergroup analyses.

**Results:**

When the novel object recognition test (NORT) results were evaluated, the Alzheimer + thymol (ALZ+TYM) group showed a significant increase in the recognition index (RI) and discrimination index (DI) compared to the Alzheimer (ALZ) group at the 24th hour. Thymol reduced working memory errors (WME), reference memory errors (RME), and maze completion time at 48, 72, and 96 hours when evaluated in terms of spatial memory in rats with Alzheimer’s disease. Furthermore, Aβ_1–42_ and ApoE levels were increased in the ALZ group compared to the control (C), while reelin and LRP8 levels were decreased in the ALZ group compared to the C group.

**Conclusion:**

The data we obtained suggest that thymol may play an effective role in cognitive processes against AD and have an anti-Alzheimer’s disease effect.

## 1. Introduction

Alzheimer’s disease (AD), the primary cause of dementia and the fifth leading cause of global mortality, affects 45 million people worldwide. Neurodegeneration in AD is characterized by abnormal accumulation of Aβ plaques, neuritic plaques, neurofibrillary tangles, and synaptic abnormalities (Hampel et al., 2021; Peng et al., 2022).

D-galactose (D-gal), a six-carbon monosaccharide found in hemicellulose, pectins, and gums, is often used as a model for studying AD mechanisms and potential treatments (Gao et al., 2016; Chroumpi et al., 2022). Abnormal D-gal metabolism leads to reactive oxygen species (ROS) production and oxidative stress, causing inflammatory aging (Gao et al., 2016).

Aluminum, a heavy metal with cholinotoxic properties, contributes to neurodegenerative disorders, including cognitive dysfunction, neurodegeneration, and apoptotic neuronal loss (Huat et al., 2019). Prolonged exposure to aluminum impairs learning ability in mice and causes neurodegenerative changes in the hippocampus, spinal cord, and cerebral cortex (Liaquat et al., 2019; Chen et al., 2021).

ApoE is responsible for cholesterol transport in the brain. There are three common isoforms in humans, ApoE2, ApoE3, and ApoE4 (Knopman et al., 2021). Heterozygous carriers of the ɛ4 allele of the gene encoding ApoE are four times more likely to develop Alzheimer’s disease (Vecchio et al., 2022). ApoE4 increases beta-amyloid aggregation, contributing to neurofibrillary tangle formation and the pathogenesis of AD through processes such as neuroinflammation and synaptic loss (Mamun et al., 2020).

Reelin, a 450 kDa extracellular glycoprotein, is crucial for brain development and maturation. It is produced by Cajal-Retzius neurons and regulates neuronal migration. In adult neurons, it controls synaptic activity, improves memory and learning abilities, and exhibits anti-Alzheimer’s disease effects by inhibiting Tau phosphorylation and reducing amyloidogenic amyloid precursor protein (APP) processing (Jossin, 2020).

LRP8, a low-density lipoprotein receptor, is highly expressed in neurons. It has 19 exons and is located on chromosome 1p34 (Ma et al., 2002). Reelin can prevent Aβ-induced neurotoxicity by binding to LRP8 (Passarella et al., 2022).

Thymol, a white crystalline monoterpene, is essential for nutrition and has antiinflammatory, antioxidant, and anticancer properties (Nagoor Meeran et al., 2017; Zengin et al., 2018; Bora et al., 2022; Şehitoğlu et al., 2023). Its lipophilic properties allow it to pass through the blood-brain barrier and affect ion channels and neurons (Zolfaghari and Vatanparast, 2020). In this study, we investigated the effect of thymol on cognitive functions and the levels of glutathione (GSH), malondialdehyde (MDA), ApoE, reelin, LRP8, and Aβ_1–42_ in an AD model induced in male Wistar Albino rats via D-gal and aluminum chloride (AlCl_3_) application.

## 2. Materials and methods

### 2.1 Chemicals

Thymol, D-gal, and AlCl_3_ were purchased from Sigma-Aldrich (St. Louis, MO, USA).

### 2.2 Animals

This study was conducted at the Üsküdar University Experimental Research Unit (ÜSKÜDAB) laboratory. Fifty male Wistar albino rats, aged 3 months, were used. The rats were kept in standard cages at a temperature of 22 ± 2 °C with a 12-h light-dark cycle and provided ad libitum access to standard pellet feed.

### 2.3. Experimental procedure

**Figure 1:**
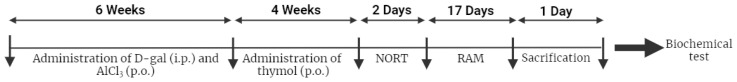
Experimental timeline.

Group 1—Control group (C); Group 2—Vehicle group (V): Administration of 0.5 mL of saline via oral gavage (p.o.) and intraperitoneally (i.p.) was carried out for 6 weeks, followed by oral administration of 0.5 mL of sunflower oil for the next 4 weeks; Group 3—Alzheimer group (ALZ): Rats received AlCl_3_ (200 mg/kg/day p.o.) and D-gal (60 mg/kg/day i.p.) dissolved in 0.5 mL of saline for 6 weeks (Zhang et al., 2016); Group 4—Thymol group (TYM): Thymol was orally administered at a dose of 30 mg/kg/day in sunflower oil for 4 weeks (Asadbegi et al., 2017); Group 5—Alzheimer + thymol group (ALZ+TYM): Rats were treated with AlCl_3_ (200 mg/kg/day p.o.) and D-gal (60 mg/kg/day i.p.) for 6 weeks, followed by oral administration of thymol at a dose of 30 mg/kg/day for 4 weeks. Experimental flow was carried out according as depicted in Figure 1.

### 2.4. Novel object recognition test (NORT)

The NORT measures rats’ short-term (ST) and long-term (LT) visual memory through four stages: habituation, retention, ST, and LT memory sessions, using identical objects of different colors and shapes.

**Figure 2:**
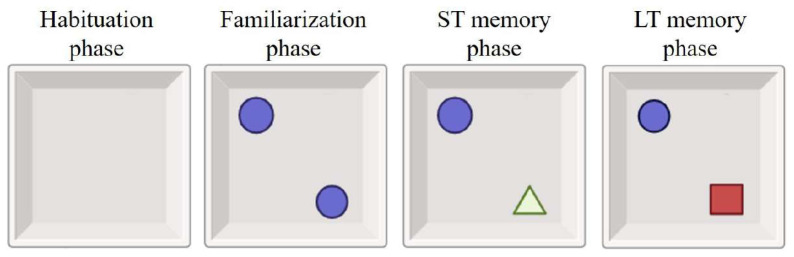
NORT experimental setup (created with BioRender.com).

During the habituation phase, the animals were accustomed to an empty apparatus and after 24 h, the familiarization phase began, in which two identical objects were placed in the apparatus. Two hours later, the ST memory phase began, wherein one object was replaced with a novel one. After 24 h, the LT memory phase began, with the replacement of the object changed in the previous phase with another novel object (Figure 2). All sessions were conducted once a day for each animal at the same time.

To prevent odor interference, objects and test apparatus were cleaned with ethyl alcohol between all tests. Rats’ discrimination index (DI) and recognition index (RI) were calculated to determine their ability to discriminate between familiar objects, suggesting that healthy animals should spend more time investigating unfamiliar things. DI and RI were calculated according to Sangüesa et al. (2018) and Diaz et al. (2021).

### 2.5. Radial arm maze (RAM)

**Figure 3:**
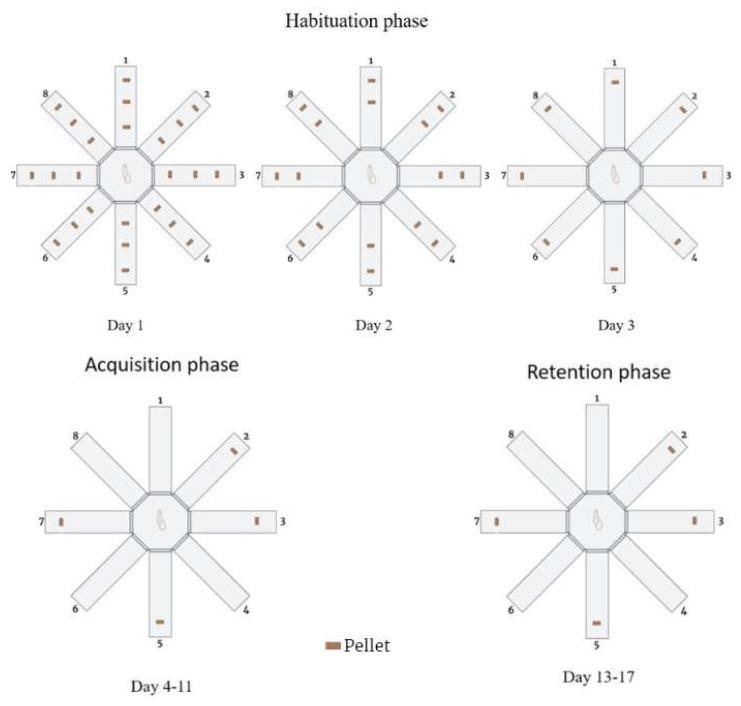
RAM experimental setup (created with BioRender.com).

Eight horizontal arms, each measuring 57 × 11 cm, arranged radially around a central platform raised 80 cm above the ground. After each measurement, the platform was cleaned with 10% alcohol. Before testing, the rats’ body weight was reduced by 15%. A food reward was placed at the end of each arm, and the test was carried out while the animals were in a hungry state. All sessions for each animal were conducted once a day at the same time. The experiment consisted of three phases (Figure 3): 1) Habituation phase, involving maze exploration trials (15 min) over 3 days. 2) Acquisition phase, which is the learning phase of the animals over 8 days (5 min). 3) Retention phase performed 48, 72, 96, 120, and 144 h after the completion of the previous session (5 min). A rat was considered to have entered an arm when all four paws crossed the arm’s entrance, and the time taken to complete the maze was recorded. Reentering a previously visited baited arm was considered a working memory error (WME), while entering a nonbaited arm was defined as a reference memory error (RME) (Valladolid Acebes et al., 2011).

### 2.6. Preparation of serum and brain tissue samples

At the end of the experimental period, Ketamine (80–100 mg/kg) and Xylazine (8–10 mg/kg) were injected intramuscularly as anesthetic agents, and heart blood samples were collected from the rats. Blood samples were centrifuged at 3000 rpm for 10 min and stored at −80 °C.

At the end of the experiment, brain tissues were quickly removed, and the cerebellum and hypothalamus were separated from the total brain. The brain was then divided into left and right hemispheres. Hemispheres were homogenized in cold phosphate buffer (pH: 7.4) after being gently rinsed in saline solution (0.9%). Subsequently, the homogenates were centrifuged at 11,000 × *g* for 15 min at 4 °C.

### 2.7. Determination of GSH levels

Following the procedures of Beutler et al. (1963) and Çoban et al. (2015), the amount of GSH in blood and a homogenized brain tissue sample was determined using the spectrophotometer at 412 nm and the compound 5,5′-dithiobis (2-nitrobenzoic acid).

### 2.8. Determination of MDA levels

Based on the study of lipid peroxidation (LPO) levels, MDA levels in serum and a homogenized brain tissue sample were assessed. LPO was calculated by detecting the concentration of MDA using thiobarbituric acid (TBA) according to Çoban et al. (2015).

### 2.9. ELISA assays

Protein levels of Aβ_1–42_, ApoE, reelin, and LRP8 were measured using kits from Shanghai Sunred Biological Technology (cat no. 201-11-0094, cat no. 201-11-0746, cat no. 201-11-1484, and cat no. 201-11-1050). The manufacturer’s protocol was followed for the experiments.

### 2.10. Statistical analysis

Statistical analysis was performed using Graphpad Prism 8.3.0 (Graphpad Prism Software, San Diego, CA). The comparison of statistical analyses between groups was determined using one-way ANOVA-LSD test, with statistical significance set at p < 0.05. Results are presented as mean ± SEM.

## 3. Results

### 3.1. Comparison of recognition memory performance of groups

**Figure 4:**
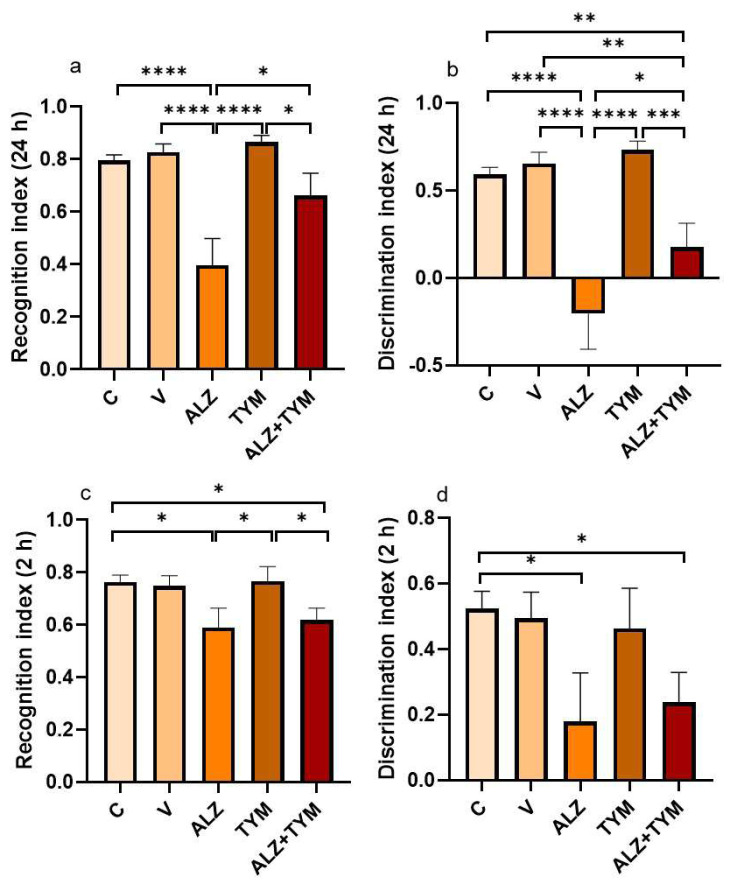
**a**. Long-term memory recognition indexes of groups, **b**. Long-term memory discrimination indexes of groups, **c**. Short-term memory recognition indexes of groups, **d**. Short-term memory discrimination indexes of groups.

As illustrated in Figure 4a, a significant decrease in the LT memory RI was found in the ALZ group when compared to the other groups. The ALZ+TYM group showed a significant decrease in LT memory RI compared to the TYM group (p = 0.0125), while a significant increase was observed in the ALZ group (p = 0.0138).

As shown in Figure 4b, a significant decrease in the LT memory DI was found in the ALZ group when compared with other groups. At the same time, it was found that the ALZ+TYM group had a decrease in the LT memory DI compared to the TYM (p = 0.0004), V (p = 0.0039), and C (p = 0.0063) groups.

As illustrated in Figure 4c, a significant decrease was found in the ST memory RI in the ALZ group when compared to the C (p = 0.0163) and TYM groups (p = 0.0159). It was found that the ALZ+TYM group had a decrease in the ST memory RI compared to the TYM (p = 0.0344) and C (p = 0.0356) groups.

As shown in Figure 4d, a significant decrease was found in the ST memory DI when the ALZ group was compared with the C (p = 0.0232) group. However, it was found that the ALZ+TYM group had a decrease in the ST memory DI compared to the C (p = 0.0490) group.

### 3.2. Comparison of spatial memory performance of groups

**Figure 5:**
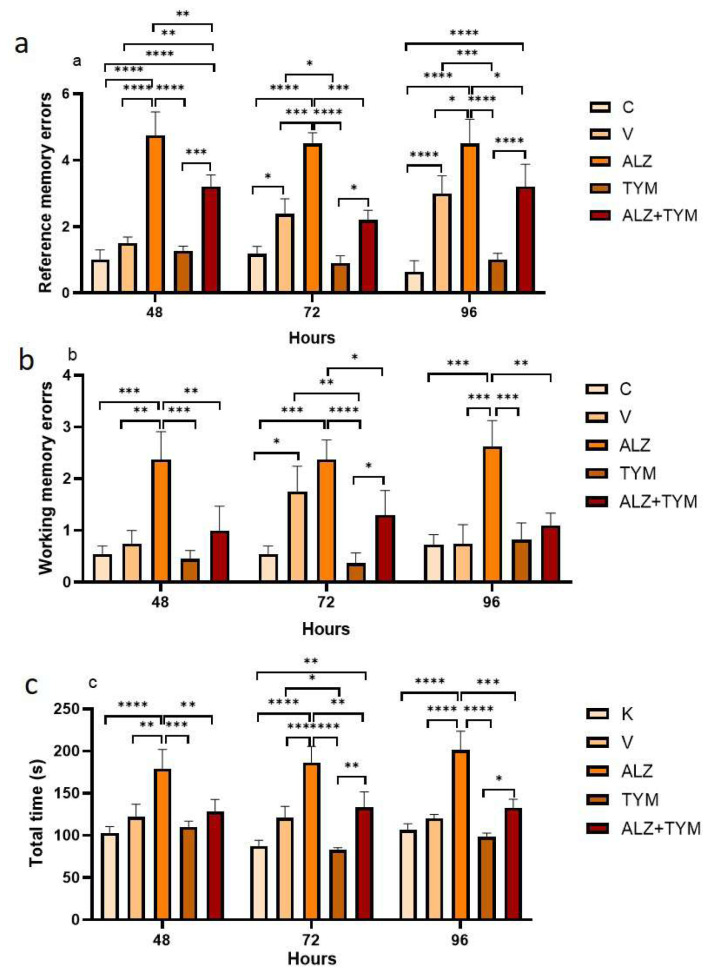
**a**. Reference memory errors of groups, **b**. Working memory errors of groups, **c**. Time for groups to finish the maze (*: p < 0.05; **: p < 0.01; ***: p < 0.001; ****: p < 0.0001).

As illustrated in Figure 5a, in 48 hours data, it was found that the ALZ group made significantly more RME when compared to other groups. Also, the ALZ+TYM group made significantly more RME compared to the C (p < 0.0001), V (p = 0.0041), and TYM groups (p = 0.0004).

In the 72 h data, it was found that the ALZ group made significantly more RME when compared to the C (p < 0.0001), V (p = 0.0007), TYM (p < 0.0001), and ALZ+TYM (p = 0.0001) groups. Also, the ALZ+TYM group made significantly more RME compared to the TYM groups (p = 0.0004). However, the V group made significantly more RME when compared to the C (p = 0.0380).

In the 96 h data, it was found that the ALZ group made significantly more RME when compared to the C (p < 0.0001), V (p = 0.0156), TYM (p < 0.0001), and ALZ+TYM (p = 0.0269) groups. Also, the ALZ+TYM group made significantly more RME compared to the C (p < 0.0001) and TYM (p < 0.0001) groups. However, the V group made significantly more RME when compared to the C (p < 0.0001) and TYM (p = 0.0006) groups.

As shown in Figure 5b, in the 48 h data, it was found that the ALZ group made significantly more WME when compared to the C (p = 0.0002), V (p = 0.0019), TYM (p < 0.0001), and ALZ+TYM (p = 0.0055) groups.

In the 72 h data, it was found that the ALZ group made significantly more WME when compared to the C (p = 0.0002), TYM (p < 0.0001), and ALZ+TYM (p = 0.0289) groups. Also, the ALZ+TYM group made significantly more WME compared to the TYM group (p = 0.0386). However, the V group made significantly more WME when compared to the C (p = 0.0127) and TYM (p = 0.0043) groups.

In the 96 h data, it was found that the ALZ group made significantly more WME when compared to the C (p = 0.0001), V (p = 0.0004), TYM (p = 0.0002), and ALZ+TYM (p = 0.0021) groups.

As illustrated in Figure 5c, in the 48 h data, the time to finish the maze was significantly longer in the ALZ group when compared to the C (p < 0.0001), V (p = 0.0030), TYM (p = 0.0001), and ALZ+TYM (p = 0.0056) groups.

In the 72 h data, the time to finish the maze was significantly longer in the ALZ group when compared to the C (p < 0.0001), V (p = 0.0008), TYM (p < 0.0001), and ALZ+TYM (p = 0.0037) groups. Also, the time to finish the maze was significantly longer in the ALZ+TYM group compared to the C (p = 0.0081) and TYM (p = 0.0027) groups. Also, the time to finish the maze was significantly longer in the V group compared to the TYM (p = 0.0302) group.

In the 96 h data, the time to finish the maze was significantly longer in the ALZ group when compared to the C (p < 0.0001), V (p < 0.0001), TYM (p < 0.0001), and ALZ+TYM (p = 0.0002) groups. Also, the time to finish the maze was significantly longer in the ALZ+TYM group when compared to the TYM (p = 0.0390).

### 3.3. Comparison of GSH-serum, GSH-brain, MDA-serum, MDA-brain parameters of the groups

**Figure 6:**
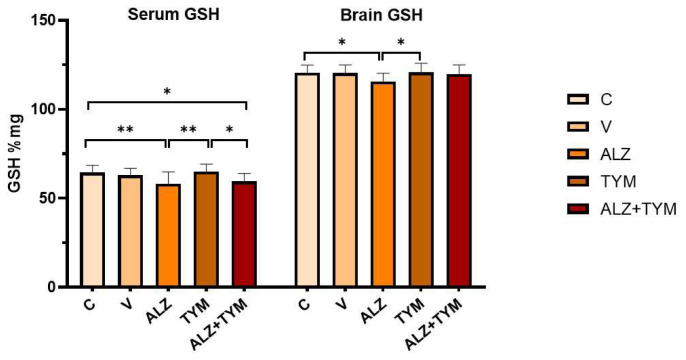
Serum and brain GSH levels of the groups (*: p < 0.05; **: p < 0.01).

As shown in Figure 6, the GSH levels of the ALZ group were significantly decreased compared to the C (p = 0.0072) and TYM (p = 0.0036) groups. The GSH levels of ALZ+TYM group were significantly decreased compared to the C (p = 0.0252) and TYM (p = 0.0129) groups. When the brain GSH levels of the groups were examined, the GSH levels of the ALZ group were significantly decreased compared to the C (p = 0.0401) and TYM (p = 0.0256) groups.

**Figure 7:**
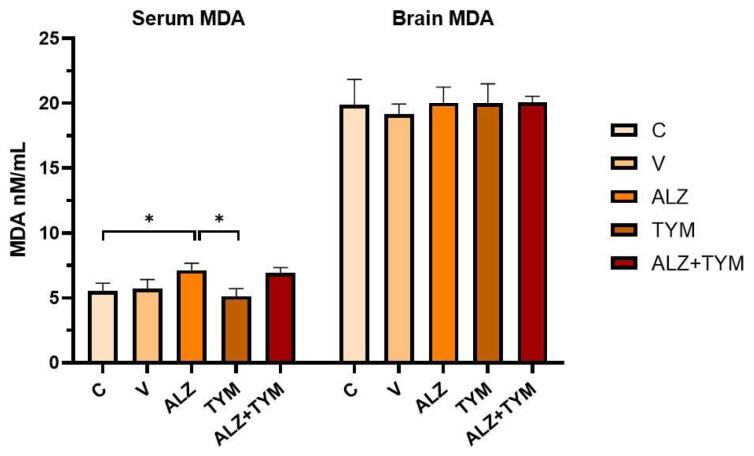
Serum and brain MDA levels of the groups (*: p < 0.05).

As illustrated in Figure 7, the serum MDA levels of the ALZ group increased significantly compared to the C (p = 0.0299) and TYM (p = 0.0206) groups. Brain MDA levels did not differ significantly between the groups. (p > 0.05).

### 3.4. Comparison of Aβ_1–42_, ApoE, LRP8, and reelin protein levels of groups

**Figure 8:**
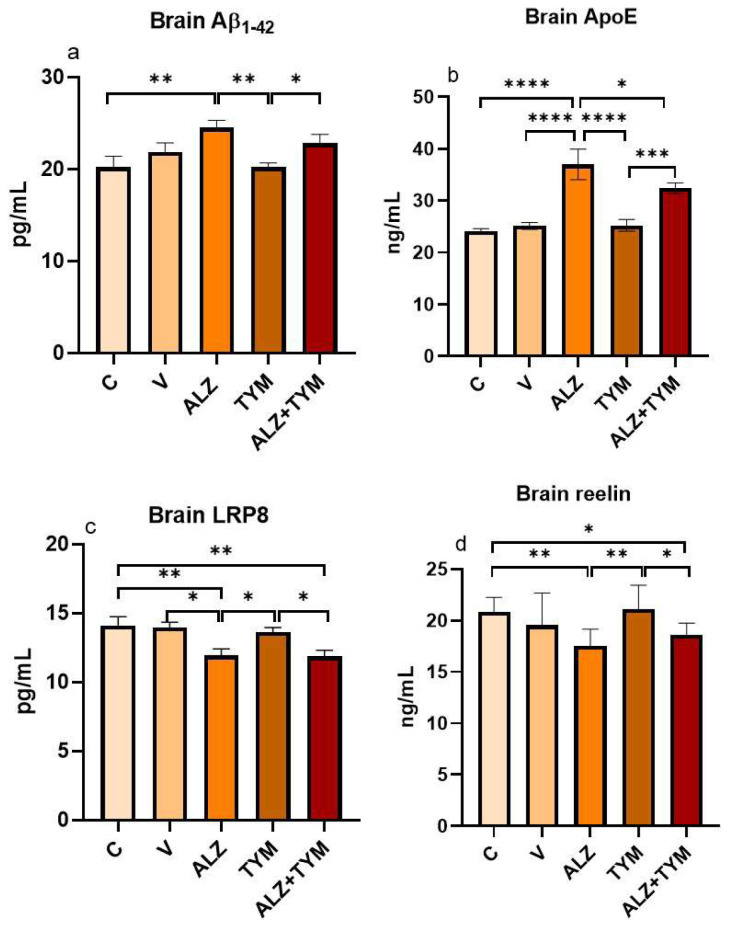
Aβ_1–42_, ApoE, LRP8, and reelin levels of the group brains (*: p < 0.05; **: p < 0.005; ***: p < 0.001; ****: p < 0.0001).

As shown in Figure 8a, the brain Aβ_1–42_ levels of the ALZ group were significantly increased compared to the C (p = 0.0022) and TYM (p = 0.002) groups. A significant increase was found in the ALZ+TYM group compared to the TYM group (p = 0.0491). It was determined that the Aβ_1–42_ levels of the ALZ+TYM group showed a slight decrease compared to the ALZ group (p > 0.05).

According to Figure 8b, the brain ApoE levels of ALZ group increased significantly compared to the C (p < 0.0001), V (p < 0.0001), TYM (p < 0.0001), and ALZ+TYM (p = 0.0328) groups. A significant increase was found in the ALZ+TYM group compared to the TYM group (p = 0.0007).

As illustrated in Figure 8c, the brain LRP8 levels of the ALZ group were significantly decreased compared to the C (p = 0.0026), V (p = 0.0117), and TYM (p = 0.0134) groups. The LRP8 levels of the ALZ+TYM group were found to be significantly decreased compared to TYM (p = 0.0105) and C (p = 0.002) groups.

As shown in Figure 8d, the brain reelin levels of the ALZ group were significantly decreased compared to the C (p = 0.0041) and TYM (p = 0.0017) groups. The reelin levels of ALZ+TYM group were found to be significantly decreased compared to TYM (p = 0.0215) and C (0.0419) groups.

## 4. Discussion

In the study, the effect of thymol on cognitive functions and the levels of GSH, MDA, Aβ_1–42_, ApoE, reelin, and LRP8 were investigated in an AD model induced in male Wistar Albino rats through D-gal and AlCl_3_ application. The obtained results were discussed and compared with the literature.

Thymol reduces the production of proinflammatory cytokines (TNF-α) by inhibiting oxidative stress markers (GSH/GSSG, H2O2, and MDA), which increase after scopolamine application. Thymol raises phospho-GSK3β (p-GSK3β), AKT, and BDNF levels (FangFang et al., 2017; Timalsina et al., 2023). Increased p-GSK3β and BDNF stimulate adult hippocampal neurogenesis and improve learning and memory. Furthermore, the Aβ_1–42_ accumulation triggered by the scopolamine-induced neuroinflammatory pathway is inhibited by p-GSK3β and BDNF (Timalsina et al., 2023). In addition, thymol increases the expression of P-Ser473 AKT and P-Ser9 GSK3β by decreasing the level of P-Ser307 IRS-1. The protective effects of thymol on cognitive disorders are associated with the upregulation of the nuclear respiratory factor (Nrf2)/heme oxygenase-1(HO-1) pathway. In conclusion, thymol exerts beneficial effects on cognitive deficits by improving hippocampal insulin resistance and activating Nrf2/HO-1 signaling (FangFang et al., 2017). Thymol has been associated with potential therapeutic benefits in preventing or modulating AD by reducing ROS production and increasing the activity of PKC, a memory-related protein (Azizi et al., 2020).

NORT is a nonrewarding test that assesses rodent cognitive status by calculating DI and RI, indicating the ability to distinguish new and familiar objects, with healthy animals spending more time exploring new objects (Bengoetxea et al., 2015). Transgenic AD animals exhibit prolonged exposure to familiar objects and lower RI compared to wild-type mice (Zhang et al., 2012), with significant reductions in DI and RI compared to the control group (Wang et al., 2023).

The study found no significant difference in RI and DI results between the ALZ+TYM group and the ALZ group at the 2nd hour, but a significant decrease compared to the C group. However, at the 24th hour evaluation, the ALZ+TYM group demonstrated a significant increase in RI and DI compared to the ALZ group.

Thymol administration has been reported to improve memory impairment and exhibit neuroprotective effects in rats (Asadbegi et al., 2017; Asadbegi et al., 2018). AlCl_3_ administration decreases spatial learning and memory in Wistar albino rats (Hamdan et al., 2022), while D-gal administration increases WME and RME in mice, indicating cognitive and spatial learning disorders (Hao et al., 2022).

The study revealed that thymol reduced WME in rats with AD at 48, 72, and 96 hours and improved ST memory in the ALZ+TYM group. However, the ALZ+TYM group did not completely improve errors compared to the C group. RME in the ALZ group increased at 48, 72, and 96 hours compared to the other groups, but a decrease was observed in RME in the ALZ+TYM group. The TYM group completed the radial arm maze faster than the ALZ group, suggesting that thymol may increase locomotor activity. Further dose trials of thymol against Alzheimer’s disease are warranted. The V group exhibited an increase in WME and RME compared to the C group, indicating the need for future research.

Streptozotocin-induced AD in Wistar albino rats showed decreased GSH levels and increased MDA levels in the cortex and hippocampus regions (Noor et al., 2022; Rajkumar et al., 2022), while AD induced by AlCl_3_ and D-gal demonstrated decreased hippocampal levels and increased MDA levels (Haider et al., 2020). The study revealed that the ALZ group had significantly lower serum GSH and brain GSH levels compared to the C group, while their serum MDA levels increased significantly compared to the C group.

Thymol has been found to decrease MDA levels in rat neurotoxicity models while increasing GSH, SOD, and catalase levels in brain tissue (Nagoor Meeran et al., 2017; Asadbegi et al., 2018). The study found that the TYM group had significantly higher serum and brain GSH levels compared to the ALZ group.

Research shows that rat models of Alzheimer’s disease (AD) induced by AlCl_3_ and D-gal lead to increased brain Aβ_1–42_ levels (Zhang et al., 2016), leading to neurotoxic oligomers, and thymol administration reduces these plaques (Asadbegi et al., 2017; Asadbegi et al., 2018). The study revealed a significant increase in brain Aβ_1–42_ levels in the ALZ group compared to the C and TYM groups.

ApoE4 contributes to AD pathogenesis through increased beta-amyloid aggregation, neuroinflammation, and synaptic loss, resulting in neurofibrillary tangle formation (Mamun et al., 2020). AlCl_3_ administration significantly increased ApoE4 levels in rats, while thymol administration decreased them (Hamdan et al., 2022). The study revealed a significant increase in brain ApoE levels in the ALZ group and ALZ+TYM group, marking the first study to investigate the impact of thymol on ApoE levels in Alzheimer’s disease.

Reelin, a protein, plays a crucial role in learning and memory by regulating NMDA receptor functions, and its overexpression can prevent neuronal death and enhance memory.

It also plays a crucial role in memory, modulating NMDA receptor functions and enhancing learning and hippocampal Long-term potentiation (LTP), while protecting against neuronal death and reducing amyloid plaque formation (Chen et al., 2005; Pujadas et al., 2014). The study revealed a significant decrease in brain reelin levels in the ALZ and ALZ+TYM groups compared to the TYM and C groups.

In vitro, in vivo, and postmortem studies have reported that LRP8 levels in AD are significantly reduced compared to healthy controls (Hinrich et al., 2016; Mata Balaguer et al., 2018). The study revealed a significant decrease in brain LRP8 levels in the ALZ and ALZ+TYM groups compared to the C and TYM groups.

In conclusion, the data we obtained suggest that thymol may play an effective role in cognitive processes against AD and have an anti-Alzheimer’s disease effect. A limitation of our study is that the levels of ApoE isoforms cannot be measured separately. These isoforms should be evaluated separately in future studies.
